# Primary hyperparathyroidism and metabolic risk factors, impact of parathyroidectomy and vitamin D supplementation, and results of a randomized double-blind study

**DOI:** 10.1530/EJE-13-0547

**Published:** 2013-12

**Authors:** Sophie Norenstedt, Ylva Pernow, Kerstin Brismar, Maria Sääf, Ayla Ekip, Fredrik Granath, Jan Zedenius, Inga-Lena Nilsson

**Affiliations:** 1 Department of Molecular Medicine and SurgeryKarolinska InstitutetStockholmSweden; 2 Department of Endocrine SurgeryKarolinska University HospitalStockholmSweden; 3 Department of Endocrinology, Metabolism and DiabetesKarolinska University HospitalStockholmSweden; 4 Department of MedicineKarolinska InstitutetStockholmSweden

## Abstract

**Background:**

Vitamin D insufficiency may increase the risk for cardio metabolic disturbances in patients with primary hyperparathyroidism (PHPT).

**Objective:**

To analyze the vitamin D status and indices of the metabolic syndrome in PHPT patients and the effect of vitamin D supplementation after parathyroid adenomectomy (PTX).

**Design and methods:**

Double-blinded, randomized clinical trial (ClinicalTrials.gov Identifier: NCT00982722) performed at Karolinska University Hospital, Sweden, April 2008 to November 2011. One hundred and fifty consecutive patients with PHPT (119 women) were randomized after PTX, 75 to oral treatment with calcium carbonate 1000 mg daily and 75 to calcium carbonate 1000 mg and cholecalciferol 1600 IU daily over 12 months. Changes in metabolic profile and ambulatory blood pressure (BP) were analyzed. Main outcome measures were changes in metabolic factors, BP, and body composition.

**Results:**

The 25-hydroxyvitamin D (25-OH-D)-level was <50 nmol/l in 76% of the patients before PTX. After PTX, glucose, insulin, and IGF1 decreased, while the 25-OH-D and the IGF-binding protein 1 increased and remained unchanged at follow-up after study medication. One year of vitamin D supplementation resulted in lower parathyroid hormone (PTH) (40 (34–52) vs 49 (38–66) ng/l) and higher 25-OH-D (76 (65–93) vs 49 (40–62) nmol/l; *P*<0.05). Other laboratory parameters were stable compared with after PTX. Systolic BP decreased and total bone mineral content increased in both groups.

**Conclusion:**

Except for the lowering of the PTH level, no additive effect of vitamin D supplementation was seen. However, PTX proved effective in reducing insulin resistance.

## Introduction

Primary hyperparathyroidism (PHPT) is associated with an increased risk of cardiovascular mortality (1, 2). Hypertension is an important risk factor in PHPT, accompanied by dyslipidemia, glucose intolerance, and insulin resistance (3, 4, 5). However, the relationship between the biochemical disturbances in PHPT and the risk of cardiovascular complications is not fully understood. In the general population, associations have been reported between circulating parathyroid hormone (PTH) levels and cardiovascular complications and mortality (6). The frequent coexistence of hypovitaminosis D and PHPT may be an important link to the metabolic syndrome (3). In population-based studies, low vitamin D levels have been associated with higher fasting glucose, higher levels of HbA1c, higher insulin resistance, higher blood pressure (BP), and a greater risk of cardiovascular complications (7, 8, 9, 10). Elevation of BP has been shown to increase the risk of cardiovascular mortality (11). Hypertension is associated with insulin resistance in at least one-third of patients (12). It has been suggested that insulin resistance is a key mediator of the association between vitamin D status and risk of the metabolic syndrome (7, 8). Insulin resistance is associated with endothelial dysfunction, hypertension, and cardiovascular complications. The insulin-like growth factor-binding protein 1 (IGFBP1) is a good marker of insulin sensitivity and cardiovascular risk (12, 13, 14, 15). IGFBPs modulate the bioactivity of the IGF. Low levels of IGFBP1 have been associated with the risk of development of diabetes (16). Increasing levels of IGFBP1 seem to have favorable effects on insulin sensitivity, hypertension, and other cardiovascular risk factors (14, 17).

After surgical cure, some patients still have elevated levels of PTH which, at least in some of the cases, could indicate a coexistent secondary hyperparathyroidism. Although some evidence has been provided that low serum 25-hydroxyvitamin D (25-OH-D) may be involved in the development of the metabolic syndrome, one has to be aware of several possible confounders like PTH, serum calcium, factors involved in the IGF1 system, physical activity, and social status (7). The close relationship between circulating concentrations of vitamin D and PTH has made it hard to establish whether hypovitaminosis D in PHPT is a causative factor or merely a marker of worse disease (18). The aim of this randomized clinical trial was to evaluate the effect of vitamin D supplementation, beyond that of parathyroid adenomectomy (PTX), on PTH levels, BP, and other cardiovascular and metabolic risk factors.

## Subjects and methods

From April 2008 to November 2010, 159 consecutive patients with PHPT subjected to PTX were included in a double-blinded clinical trial (ClinicalTrials.gov Identifier: NCT00982722).

### The indications for surgery

**Figure 1:**
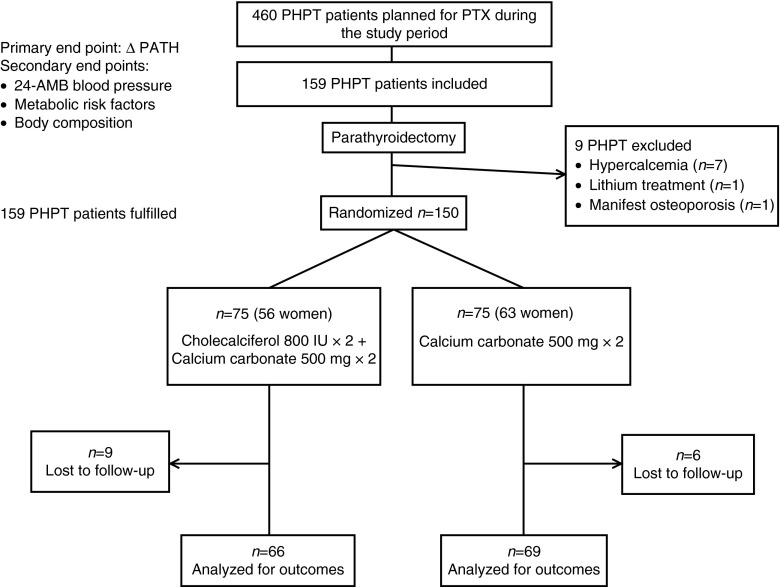
Flow chart of the study.

In total, 460 PHPT patients were subjected to PTX during the study period at our clinic (Fig. 1). Exclusion criteria were age under 18, manifest osteoporosis at PHPT diagnosis, persistent hypercalcemia after surgery, postoperative hypocalcemia requiring vitamin D treatment, glomerular filtration rate (GFR) <40 ml/min, pregnancy, breast feeding, or logistical difficulties, for example living far from the hospital. We did not exclude patients with cardiovascular risk factors. However, patients with insulin treatment (*n*=2) were excluded from the analyses of glucose, insulin, and homeostatic model assessment insulin resistance (HOMA-IR). Included patients had to withdraw any current supplementation with calcium and vitamin D during the study period. Patients on vitamin D treatment prescribed for medical reasons were not included in the study. Nine patients were excluded before randomization (Fig. 1). Six±two weeks after PTX, 150 patients were randomized, 75 into each group, to 1 year of oral treatment with either calcium carbonate 1 g (500 mg twice daily) alone (D− group) or calcium carbonate 1 g (500 mg×2)+cholecalciferol 1600 IU (800 IU twice daily) (D+ group) (Fig. 1). All tablets were identical in appearance, the tins were numbered and randomization followed a list compiled by an independent clinical research support organization. The time for randomization was chosen to make sure that the PHPT patients were cured before starting the study medication. Nearly three-quarters of the patients were randomized within 6 weeks after PTX. The primary end point was the change in PTH after PTX and treatment with the study medication. Secondary end points were vitamin D levels, insulin resistance, BP, and other cardiovascular risk factors.

The baseline characteristics (preoperatively) are presented in Table 1. Except for a higher percentage of treatment with diuretics and statins in the D− group, the groups did not differ. The patients were not stratified according to baseline 25-OH-D during randomization. One hundred and thirty-five patients had a complete follow-up. The 15 patients who dropped out were followed for a median of 6 months (min–max 1–9 months). Reasons for termination were the patient's own will (11), emigration (1), deceased (2), and symptomatic vitamin D deficiency (1). Baseline data did not differ between dropouts and those who completed the study (data not shown). BMI was calculated at baseline as weight (kg) divided by the square of height (m). Estimated renal function (GFR ml/min per 1.73 m^2^) was derived by Cockroft-Gault's formula (19).

Each patient gave written consent to participate in the study, which was approved by the local Ethics Committee, Regionala etikprövningsnämnden, EPN, Stockholm, Sweden and the Medical Products Agency of Sweden.

### Ambulatory BP

Ambulatory BP monitoring (24-h ABP) was performed with a standardized ABP device (Meditech ABPM-04 monitor, PMS Instruments, Maidenhead, UK) that was applied around the patient's nondominant arm. Patients were instructed to continue their usual daily activities while wearing the device. Patients with antihypertensive medication were instructed to continue treatment. Daytime was defined as the time from wakening to bedtime (0700–2300 h in most cases) and nighttime as the time the study participant spent in bed. The ambulatory device was set to record ABP and heart rate (HR) at 30-min intervals during daytime and 60-min intervals during nighttime. If the recording failed, a new measurement was automatically done after 2 min. One hundred and twenty-five patients completed pre-and postoperative AMB.

### Body composition

Total body mineral content (BMC), lean body mass (LBM), and fat % were estimated in a subgroup of patients (*n*=122) using dual energy X-ray absorptiometry (DXA). The same instrument (Lunar Prodigy Advance, #PA+41562, GE Healthcare) was used for every patient.

### Biochemical methods

Blood and urine samples were collected after an overnight fast at 6±2 weeks before surgery, at randomization, and after 6 and 12 months of treatment. Serum concentrations of IGF1, insulin, and plasma concentration of intact PTH were determined with electrochemiluminescence immunoassay on the Modular E system (Roche Diagnostics GmbH). Serum ionized calcium was analyzed on ABL 800 (Radiometer, Copenhagen, Denmark). Plasma concentrations of phosphate, creatinine, glucose, total cholesterol, triglycerides (TG), HDL, and LDL were measured using the Synchron LX 20 system (Beckman Coulter, Inc., Brea, CA, USA). In order to minimize inter-assay variation, the pre- and postoperative samples of 25-OH-D, IGF1, and IGFBP1 were analyzed in the same series on serum previously frozen at −70 °C. Serum concentrations of 25-OH-D were measured by chemiluminescence on the Liason XL (DiaSorin, Inc., Stillwater, MN, USA); inter-assay %CV is 4.6% at 15.5 nmol/l and 2.7% at 68.3 nmol/l and intra-assay %CV is 4.4% at 15.5 nmol/l and 2.6% at 68.3 nmol/l. Values below 50 nmol/l were considered to represent vitamin D insufficiency (20).

An in-house RIA according to the method of Povoa *et al*. (21) determined IGFBP1 concentrations in serum. The sensitivity of the RIA was 3 μg/l and the intra- and inter-assays CV were 3 and 10% respectively.

Estimates of insulin resistance were calculated using the HOMA-IR: insulin resistance=fasting glucose×fasting insulin/22.5 after conversion of insulin levels from pmol/l to μU/ml by multiplication with a factor 6.945 (22).

### Statistical analysis

The size of the cohort was determined by a power analysis. Delta PTH was chosen as the primary end point because no data on the effect of vitamin D supplementation on metabolic risk factors after parathyroid surgery were available (23, 24). The size of the study population was estimated to guarantee a power level of 80% at a confidence level of 95%. Statistical analysis was performed with the IBM SPSS Statistics version 20. As data did not follow a normal distribution, they are expressed as median and interquartile range (IQR). For comparisons between groups, the Mann–Whitney *U* test for unpaired data was used; the Kruskal–Wallis one-way ANOVA was used for comparisons with respect to independent categorical variables with more than two levels and *χ*^2^ tests for comparisons of the distributions of categorical variables. Bivariate associations between continuous variables were assessed with Spearman's *ρ* correlation test. All tests were done using two-tailed test, and *P*<0.05 was considered to be statistically significant.

## Results

The baseline data are presented in Table 2. Vitamin D insufficiency, defined as 25-OH-D <50 nmol/l, was present at baseline in 76% of the patients. Patients with 25-OH-D in the lowest quartile (<31 nmol/l) had higher levels of fP-PTH, fP-glucose, fP-insulin, HOMA-IR, and fS-TGs (Table 2). The prevalence of vitamin D insufficiency was similar among men and women.

Six weeks after PTX, the ionized calcium level was normalized in all patients (Table 3). PTH was still elevated in 50% of the patients, of whom 62/75 (84%) had a preoperative 25-OH-D <50 nmol/l compared with 68% (51/75) in the group with normal PTH (*P*=0.02).

Plasma glucose, insulin, HOMA-IR, and IGF1 decreased after PTX, while IGFBP1 increased. The changes were not correlated with the elevation of vitamin D. ΔIGF1 was inversely correlated with baseline ionized calcium and PTH levels (*r*=−0.28 and *r*=−0.26, *P*<0.01). The increase in IGFBP1 correlated with the decrease in insulin (*r*=−0.26; *P*=0.002) and HOMA-IR (*r*=−0.25; *P*=0.002).

Randomization: patients with vitamin D insufficiency and PTH-elevation after PTX were equally distributed between D+ and D−; 35 in D− and 39 in D+ (*P*=0.31) and the baseline 25-OH-D did not differ between the D+ and the D− groups. The study medication was well tolerated. One hundred and thirty-five patients completed the whole study period; 66 in D+ and 69 in D− (Tables 3 and 4).

At follow-up 1 year after randomization, D+ patients had a higher serum concentration of 25-OH-D, lower PTH, and lower IGF1 (Table 4). No other differences were observed. The total calcium concentration was within the normal reference range in all cases. Only two patients in the D+ group had a 25-OH-D-level below 50 nmol/l compared with 36 patients in the D− group (*P*<0.001). ΔPTH was inversely correlated with Δ25-OH-D (*r*=−0.37; *P*<0.001). In 26 cases, PTH was still above the reference range (65 ng/l). Twelve patients with PTH elevation (46%) had a 25-OH-D-level below 50 nmol/l, all in the D− group; one patient had an ionized calcium concentration above the normal range (1.34; 1.15–1.33). HOMA-IR and the serum levels of IGF1, IGFBP1, and insulin were unchanged compared with after PTX in both groups. In the whole cohort, the change in insulin resistance between baseline and after 1 year of study medication was inversely correlated with changes in IGFBP1 (*r*=−0.28; *P*=0.001) and directly correlated with changes in IGF1 (*r*=0.22; *P*=0.01). A correlation between ΔHOMA-IR and ΔBMI (*r*=0.22; *P*=0.015) was found but no correlation to ΔPTH, Δ25-OH-D, or Δionized calcium.

We divided the patients with and without 25-OH-D <50 nmol/l into two subgroups and compared D+ and D− within each subgroup. The result did not differ from the analysis of the whole group of 150 patients; vitamin D supplementation lowered PTH and gave higher 25-OH-D levels in D+, but had no other effect in either subgroup (data not shown).

The different outcomes in D+ and D− at 1-year follow-up were also compared by means of regression models. First, we adjusted for age and BMI at randomization as continuous variables. In a second model, we further adjusted for the outcome measurement at randomization by the analysis of covariance (ANCOVA). The results were the same after adjustments.

ABP was monitored before PTX and after 1 year of study medication (Table 5). Median 24-h systolic BP (SBP) at baseline was significantly correlated with baseline PTH (*r*=0.24), serum insulin (*r*=0.30), and TG (*r*=0.37), *P*<0.01 for all, and inversely correlated with IGFBP1 (*r*=−0.19; *P*<0.05): 24-h SBP decreased in both groups. The change in 24-h SBP was not correlated with changes in PTH, ionized calcium, or 25-OH-D (data not shown). Eleven patients equally distributed between the D+ and the D− group were able to either cease or reduce their antihypertensive treatment. Vitamin D supplementation did not give any additive effect.

Body composition was evaluated in a subgroup of patients (*n*=122; equally distributed between D+ and D−) (Table 5). Total BMC increased in both D+ and D−. There was an inverse correlation between ΔBMC and ΔPTH (*r*=−0.30, *P*=0.002) but no correlation to Δ25-OH-D or Δionized calcium.

## Discussion

To summarize, 76% of patients had a vitamin D concentration below the cutoff level (50 nmol/l) recommended for vitamin D substitution by international guidelines (18, 20). Improvements in insulin resistance, BP, and whole-body mineral density were observed 12 months after PTX but were not related to the vitamin D status. Except for lowering the PTH level, no additive effect of vitamin D supplementation was seen. Although the vitamin D supplementation was well tolerated and successful in reaching an adequate concentration of 25-OH-D in serum, the improvements in risk factors connected with the metabolic syndrome were not related to the vitamin D concentration.

Our data is in agreement with recently published meta-analyses. Thus, despite an increased risk of cardiovascular events in patients with 25-OH-D levels below 37.5 nmol/l in large population-based studies, no convincing effect of vitamin D supplementation has been shown (25, 26). The exact nature of the relationship between hypovitaminosis D and risk factors associated with the metabolic syndrome remains unclear and one has to be aware of several confounders complicating the relation (7). We still lack globally accepted cutoff levels for vitamin D deficiency and insufficiency (20). Valcour *et al*. (18) found an ongoing decline in PTH levels at 25-OH-D <75 nmol/l and one may speculate that a low circulating 25-OH-D concentration may in fact stand for an epiphenomenon coupled with other risk factors, for example, secondary hyperparathyroidism. In the general population, PTH in the upper normal range has been associated with increased risk of cardiovascular complications (6, 27). We cannot exclude that the higher PTH concentration in the D− group brings negative effects in the long term.

The positive results from this study regarding improvements in insulin resistance were observed after PTX and before the start of study medication. Neither could the minor but significant improvements in BP and total bone mineral content be related to vitamin D status, although the supplementation with calcium carbonate may influence the results (28). In a large population-based observational study, higher 25-OH-D levels but not higher dietary calcium intake was associated with decreased risk of developing diabetes (8). In an experimental setting, the insulin sensitivity, measured by euglycemic–hyperinsulinemic clamp in a population-based cohort of elderly men, decreased when the serum calcium concentration increased (29). Available reports on the relationship between PHPT and insulin resistance are sparse and contradictory (30, 31, 32). The simultaneous reduction in glucose, insulin, and IGF1 and the increase in IGFBP1 seen postoperatively, and remaining at follow-up, strengthen the possible reversibility of the metabolic syndrome coupled with PHPT. IGFBP1 is a marker of insulin sensitivity. Low levels are seen in insulin resistance and hypertension and are associated with increased cardiovascular risk (12, 14, 16). Life style interventions increase IGFBP1 and were associated with increased insulin sensitivity (17).

In a study based on two populations, the IGFBP1 concentration was inversely correlated with several established cardiovascular risk factors and directly correlated with insulin sensitivity (14). The correlation between the postoperative increase in IGFBP1 and the decrease in IGFI and in PTH that we observed in our study could indicate a connection with PTH-concentration. However, there was no difference in the IGFBP1 concentration between the D+ and D− groups at 1-year follow-up, despite significantly lower PTH in the D+ group.

Control of hypertension appears to be crucial in the prevention of cardiovascular complications. The isolated decrease in SBP found in this study may hypothetically be related to increased insulin sensitivity and increased vascular resistance, mediated by a disease-related inotropic effect that may result in endothelial dysfunction and an increased vascular resistance. In an earlier experimental study, we observed a dose-related impairment in endothelial vasodilatory function and elevation of SBP during systemic infusion of calcium in healthy subjects (33). Our findings are consistent with the results from a previous case–control study, restricted to subjects without known cardiovascular risk factors, in which the PHPT patients had slightly higher SBP compared with controls and both SBP and regional systolic myocardial velocities decreased significantly after PTX (34). The Framingham Study has confirmed that the risk of cardiovascular complications increases incrementally with BP even within the normal range (35). An increase in BP from optimal <120/80 to 130–139/85–89 resulted in an almost twofold risk of cardiovascular consequences (11). The Framingham Study has also demonstrated that SBP is a more important risk factor than DBP (11, 35). It is also well established that 24-h ABP is superior to single office measurements in predicting a risk of cardiovascular morbidity and mortality (36). The available information on 24-h ABP in PHPT is limited to a few studies with contradictory results (37, 38, 39, 40, 41). Recently, Luigi *et al*. (37) compared patients with PHPT with patients with essential hypertension and healthy subjects, with 30 in each group. They found a strong correlation between PTH and SBP and a high prevalence of metabolic syndrome, with significant improvements after parathyroid surgery. Others have also reported a high percentage of alterations in the normal circadian rhythm of 24-h ABP in PHPT (38, 39). Despite a high proportion of vitamin D insufficiency in our PHPT cohort, we found no correlation between vitamin D levels and 24-h ABP. Most of the analyzed PHPT cohorts contained a relatively high proportion of patients with hypertensive medication, which may bias the results. The association with other cardiovascular risk factors such as diabetes often complicates the interpretation. Reversible endothelial dysfunction appears to precede structural changes (42, 43). Patients with PHPT combined with other cardiovascular risk factors seem to be more prone to arterial stiffness and atherosclerosis (44). The combination of high PTH and insulin resistance could potentiate the risk of cardiovascular complications. The clinical importance of the high percentage of postoperative PTH elevation is interesting and cannot be explained by vitamin D insufficiency alone. Persistent disease is unlikely because the patients remained normocalcemic despite later supplementation with calcium carbonate in a relatively high dose. Neither hypertension nor PTH levels are currently covered by the guidelines for treatment of asymptomatic PHPT (20). The prognostic significance of preoperative PTH levels for the reversibility of hypertension and cardiovascular complications has to be further evaluated in prospective studies and there may be reasons to reevaluate the guidelines.

The strengths of this interventional study are the randomized and double-blinded design, the close and standardized follow-up, the compatibility, and the achievement of adequate vitamin D levels, allowing sufficient statistical power. The study medication was designed specifically for the study. The use of calcium carbonate instead of placebo is a limitation, as we cannot definitely exclude that the calcium supplementation interfered with the results. However, except for the improvements in BP and total BMC, the changes were detected before the start of the study medication and remained stable during the study period. Another limitation may be the time interval between operation and randomization. We chose to randomize the patients then, to make sure that they were cured before starting the study medication. A shorter interval might have been favorable, and we cannot exclude that a potential additive effect of vitamin D may be dampened by a significant early postoperative effect. There is also a possibility that the higher percentage of patients treated with statins and diuretics in the vitamin D group might have affected the cardiovascular outcomes.

In conclusion, except for lowering the PTH level, no additive effect of vitamin D supplementation after PTX was seen. PTX proved to be effective in reducing insulin resistance. Improvements in BP and BMC were registered without coupling with vitamin D status. Our findings support a positive effect of PTX on insulin resistance, but vitamin D supplementation after PTX did not further influence the studied metabolic parameters.

## Author contribution statement

Study design: S Norenstedt, Y Pernow, J Zedenius, M Sääf, F Granath, I-L Nilsson. Study conduct: S Norenstedt, Y Pernow, J Zedenius, M Sääf, I-L Nilsson. Data collection: S Norenstedt, Y Pernow, J Zedenius, K Brismar, A Ekip, M Sääf, I-L Nilsson. Data analysis: S Norenstedt, I-L Nilsson, A Ekip, F Granath. Data interpretation: S Norenstedt, Y Pernow, J Zedenius, F Granath, K Brismar, A Ekip, I-L Nilsson. Drafting manuscript: S Norenstedt, I-L Nilsson, Y Pernow. Revising manuscript content: S Norenstedt, Y Pernow, J Zedenius, A Ekip, K Brismar, M Sääf, F Granath, I-L Nilsson. Approving final version of manuscript: S Norenstedt, Y Pernow, J Zedenius, K Brismar, A Ekip, M Sääf, F Granath, I-L Nilsson. S Norenstedt, I-L Nilsson and Y Pernow took responsibility for the integrity of the data analysis.

## Figures and Tables

**Table 1:** Clinical characteristics.

	***n*=150**	**D+**	**D−**	***P***
Age (years, median (min–max))	60 (30–80)	62 (30–78)	61 (37–80)	NS
Women/men (*n*)	119/31	58/17	61/14	NS
Women ≤50 years (*n*)	19	10	9	NS
BMI (kg/m^2^, median (min–max))	26 (17–44)	26 (17–44)	26 (18–41)	NS
Waist (cm)	94 (66–141)	95 (69–141)	92 (66–126)	NS
Weight of adenoma (mg, median (min–max))	450 (75–27 800)	500 (75–9800)	385 (92–27 800)	NS
Multiglandular disease (*n*)	4	2	2	NS
Vitamin D <50 nmol/l (*n* (%))	114 (76%)	59	55	NS
Osteoporosis (*n* (%))	69 (46%)	30	39	NS
Smokers (*n* (%))	23 (15%)	10	13	NS
Diabetes (*n* (%))	8 (5%)	4	4	NS
Antihypertensive treatment	67 (45%)	34	33	NS
Diuretics	26 (17%)	7	19	<0.05
ACE inhibitors	31 (21%)	15	16	NS
Betablockers	32 (21%)	16	16	NS
Calcium channel blockers	16 (11%)	9	7	NS
Other relevant medication				
Statins	24 (16%)	8	16	<0.05
Steroids	3 (2%)	1	2	NS
Oestrogen, systemic	6 (4%)	2	4	NS
Insulin	2 (1%)	0	2	NS
Oral antidiabetics	6 (4%)	3	3	NS

**Table 2:** Biochemistry at baseline (before surgery) in groups of different vitamin D levels, based on quartiles (I=1st quartile, II=2nd and 3rd quartile, III=4th quartile).

	**I** (*n*=39; 33♀)		**II** (*n*=74; 57♀)		**III** (*n*=37; 29♀)		***P***		
	≤31 nmol/l		32–49 nmol/l		>49 nmol/l		**I–II–III**	**I–II**	**I−(II+III)**
	Median	IQR	Median	IQR	Median	IQR	K–W	M–W	M–W
Age (years)	**62**	51–67	**60**	53–68	**63**	55–70	0.377	0.412	0.716
BMI (kg/m^2^)	**27.0**	24.0–31.0	**26.3**	23.9–29.0	**25.3**	22.4–27.3	0.234	0.467	0.250
Waist (cm)	**98.8**	88.0–109.8	**95.0**	84.7–102.0	**87.0**	80.9–96.5	0.021	0.153	0.045
Weight of adenoma (mg)	**523**	327–888	**484**	235–969	**287**	210–663	0.088	0.565	0.202
S-25-OH-D (nmol/l)	**26**	21–30	**40**	36–43	**54**	52–61	<0.001	<0.001	<0.001
P-PTH (ng/l)	**128**	104–161	**116**	89–144	**103**	84–126	0.024	0.065	0.018
S-Ca^2^^+^ (mmol/l)	**1.43**	1.38–1.46	**1.43**	1.40–1.47	**1.44**	1.37–1.49	0.792	0.477	0.497
P-phosphate (mmol/l)	**0.86**	0.69–0.92	**0.82**	0.74–0.92	**0.84**	0.78–0.93	0.913	0.901	0.963
P-creatinine (μmol/l)	**60**	52–70	**66**	57–80	**69**	62–81	0.010	0.024	0.004
P-glucose (mmol/l)^a^	**5.4**	5.1–6.3	**5.2**	5.0–5.6	**5.0**	4.7–5.4	0.006	0.054	0.010
S-insulin (pmol/l)^a^	**79**	56–129	**67**	43–98	**53**	36–70	0.009	0.091	0.016
HOMA-IR^a^	**2.7**	1.9–5.2	**2.3**	1.4–3.3	**1.7**	1.1–2.6	0.004	0.062	0.008
S-IGF1 (μg/l)	**146**	116–161	**145**	121–182	**156**	120–196	0.316	0.373	0.190
S-IGFBP1 (μg/l)	**28**	21–45	**28**	19–49	**40**	23–62	0.100	0.863	0.354
P-HDL (mmol/l)	**1.4**	1.1–1.8	**1.4**	1.3–1.7	**1.5**	1.2–2.0	0.427	0.401	0.239
P-LDL (mmol/l)	**3.8**	2.7–4.1	**3.5**	2.9–4.2	**3.4**	2.5–4.0	0.293	0.888	0.488
P-triglycerides (mmol/l)	**1.3**	0.9–1.8	**0.9**	0.8–1.3	**0.8**	0.6–1.2	0.001	0.017	0.001

K–W, Kruskal–Wallis test for unpaired data; M–W, Mann–Whitney *U* test for unpaired data; 25-OH-D, 25-hydroxyvitamin D; PTH, parathyroid hormone; Ca^2^^+^, ionized calcium; HOMA-IR, homeostatic model assessment insulin resistance; IGF1, insulin-like growth factor 1; IGFBP1, IGF binding protein 1. Normal range for each test is specified in Table 3.

a Two patients with insulin treatment were excluded from the analysis.

**Table 3:** Biochemical parameters before and after parathyroid adenomectomy (PTX).

	**Baseline**		**After PTX**		
	Median	IQR	Median	IQR	*P* (W)
S-25-OH-D (75–250 nmol/l)	**40**	31–49	**42**	33–54	0.004
P-PTH (10–65 ng/l)	**116**	89–145	**65**	53–68	<0.001
S-Ca^2^^+^ (1.15–1.33 mmol/l)	**1.43**	1.39–1.43	**1.25**	1.22–1.27	<0.001
P-phosphate (0.75–1.4 mmol/l)	**0.83**	0.74–0.92	**1.0**	0.92–1.1	<0.001
P-creatinine (♀<90, ♂<100 μmol/l)	**65**	56–76	**67**	58–75	0.400
GFR creatinine (ml/min)	**97**	79–117	**95**	79–115	0.900
P-glucose (4.0–6.0 mmol/l)^a^	**5.2**	4.9–5.6	**5.2**	4.8–5.6	0.022
S-insulin (18–173 pmol/l)^a^	**66**	43–97	**58**	37–95	<0.001
HOMA-IR^a^	**2.2**	1.4–3.3	**1.8**	1.2–3.2	<0.001
S-IGF1 (110–270 μg/l)	**144**	117–179	**138**	115–172	<0.001
S-IGFBP1	**30**	21–49	**37**	21–54	0.046
S-cholesterol (3.3–7.8 mmol/l)	**5.6**	4.8–6.1	**5.5**	4.9–6.3	0.134
P-HDL (♀01.0–2.7, ♂0.8–2.1 mmol/l)	**1.4**	1.2–1.8	**1.4**	1.2–1.8	0.825
P-LDL (1.4–5.3 mmol/l)	**3.5**	2.8–4.1	**3.5**	2.8–4.1	0.161
S-triglycerides (0.45–2.6 mmol/l)	**0.98**	0.75–1.40	**0.96**	0.70–1.52	0.256

W, Wilcoxon signed-rank sum test for paired data; 25-OH-D, 25-hydroxyvitamin D; PTH, parathyroid hormone; Ca^2^^+^, ionized calcium; GFR, glomerular filtration rate; HOMA-IR, homeostatic model assessment insulin resistance; IGF1, insulin-like growth factor 1; IGFBP1, IGF-binding protein 1.

a Patients with insulin treatment were excluded from the analysis.

**Table 4:** Biochemical parameters at randomization and after 1 year of study medication.

	**D+** (*n*=66; 48 women)					**D−** (*n*=69; 57 women)					**D+ vs D−**	
	Randomization		One year			Randomization		One year			*P* (M–W)	
	Median	IQR	Median	IQR	*P*(W)	Median	IQR	Median	IQR	*P*(W)	Randomization	One year
S-25-OH-vitD (nmol/l)	**40**	33–52	**76**	65–93	<0.001	**45**	35–54	**49**	40–62	<0.001	0.285	<0.001
P-PTH (ng/l)	**67**	52–88	**40**	34–52	<0.001	**64**	56–80	**49**	38–66	<0.001	0.610	0.011
S-Ca^2^^+^ (mmol/l)	**1.24**	1.21–1.27	**1.26**	1.23–1.28	0.07	**1.25**	1.22–1.27	**1.26**	1.22–1.28	0.028	0.872	0.928
P-phosphate (mmol/l)	**1.00**	0.94–1.10	**1.00**	0.90–1.10	0.469	**1.00**	0.90–1.10	**1.00**	0.94–1.20	0.017	0.625	0.606
P-creatinine (μmol/l)	**66**	58–74	**66**	59–78	0.07	**67**	58–77	**68**	58–80	0.011	0.462	0.536
P-glucose (mmol/l)^a^	**5.2**	4.8–5.7	**5.1**	4.8–5.6	0.891	**5.1**	4.9–5.5	**5.2**	4.8–5.5	0.526	0.386	0.747
S-insulin (pmol/l)^a^	**58**	40–106	**53**	33–81	0.807	**53**	33–88	**51**	35–82	0.114	0.216	0.852
HOMA-IR^a^	**1.9**	1.3–1.9	**1.9**	1.2–3.6	0.512	**1.8**	1.1–3.1	**1.7**	1.1–3.0	0.445	0.214	0.314
S-IGF1 (μg/l)	**132**	109–157	**129**	109–160	0.690	**139**	119–174	**149**	124–183	0.370	0.237	0.047
S-IGFBP1 (μg/l)	**33**	20–51	**34**	22–54	0.720	**43**	24–61	**38**	26–67	0.176	0.100	0.099
P-HDL (mmol/l)	**1.4**	1.2–1.9	**1.5**	1.2–1.8	0.546	**1.4**	1.2–1.8	**1.7**	1.3–1.9	0.038	0.512	0.115
P-LDL (mmol/l)	**3.7**	3.0–4.3	**3.6**	2.9–4.2	0.478	**3.4**	2.8–4.0	**3.3**	2.5–4.1	0.118	0.216	0.270
S-triglycerides (mmol/l)	**1.00**	0.74–1.70	**0.95**	0.82–1.40	0.926	**0.94**	0.60–1.45	**0.91**	0.66–1.25	0.381	0.175	0.076
Ambulatory BP (mmHg)			*n*=65					*n*=66				
24-h												
SBP	**132**	124–145	**131**	124–141	0.087	**124**	117–131	**122**	114–130	0.322	<0.001	<0.001
DBP	**78**	69–82	**75**	71–80	0.293	**73**	67–78	**72**	67–77	0.512	0.009	0.014
Daytime												
SBP	**139**	128–152	**135**	129–144	0.036	**130**	123–140	**126**	120–135	0.004	0.001	<0.001
DBP	**81**	75–88	**80**	75–84	0.246	**79**	72–83	**77**	71–84	0.198	0.031	0.040
Nighttime												
SBP	**121**	111–134	**122**	111–133	0.172	**110**	103–123	**111**	103–120	0.651	<0.001	<0.001
DBP	**68**	60–72	**66**	62–73	0.987	**63**	57–69	**63**	58–68	0.310	0.011	0.025

W, Wilcoxon signed-rank sum test for paired data; M–W, Mann–Whitney *U* test for unpaired data; 25-OH-D, 25-hydroxyvitamin D; PTH, parathyroid hormone; Ca^2^^+^, ionized calcium; GFR, glomerular filtration rate; HOMA-IR, homeostatic model assessment insulin resistance; IGF1, insulin-like growth factor 1; IGFBP1, IGF-binding protein 1; BP, blood pressure; SBP, systolic blood pressure; DBP, diastolic blood pressure. Normal range for each test is specified in Table 3.

a Patients with insulin treatment were excluded from the analysis.

**Table 5:** Changes in blood pressure and body composition from baseline to 1 year after parathyroid adenomectomy.

	**All** (*n*=129)			**D+** (*n*=64)			**D−** (*n*=65)			
	Baseline – 1 year			Baseline – 1 year			Baseline – 1 year			D+ vs D−
	Median	IQR	*P* (W)	Median	IQR	*P* (W)	Median	IQR	*P* (W)	*P* (M–W)
ΔBMI (kg/m^2^)	**0.2**	−0.1 to 0.7	0.004	**0.3**	0.02 to 1.0	0.001	**0.1**	−0.3 to 0.4	0.664	0.008
Δ24-AMB (mmHg)										
ΔMean daytime										
SBP	**−3**	−10 to 3	<0.001	**−3**	−12 to 5	0.036	**−2**	−8 to 2	0.004	0.919
DBP	**−1**	−5 to 3	0.081	**−1**	−7 to 3	0.246	**−1**	−4 to 3	0.198	0.972
ΔMean nighttime										
SBP	**−1**	−7 to 6	0.522	**−3**	−8 to 6	0.172	**1**	−6 to 7	0.651	0.179
DBP	**1**	−5 to 5	0.445	**1**	−5 to 4	0.987	**1**	−3 to 6	0.310	0.586
ΔBody composition	*n*=106			*n*=54			*n*=52			
ΔBMC (g)	**62**	−30 to 122	<0.001	**68**	−16 to 127	<0.001	**56**	−32 to 108	0.013	0.250
ΔLBM (g)	**406**	−643 to 1314	0.013	**543**	−934 to 1338	0.270	**236**	−480 to 1318	0.032	0.747
ΔFat %	**0.1**	−1.3 to 1.5	0.722	**0.3**	−1.1 to 2.3	0.158	**−0.1**	−1.5 to 1.0	0.225	0.121

W, Wilcoxon signed-rank sum test; M–W, Mann–Whitney *U* test; 24-AMB, 24-h ambulatory blood pressure; SBP, systolic blood pressure; DBP, diastolic blood pressure; BMC, bone mineral content; LBM, lean body mass.
